# Low vitamin D status and 10-year dementia risk in sensory-impaired adults: a propensity score-matched cohort study

**DOI:** 10.3389/fnut.2026.1822195

**Published:** 2026-03-31

**Authors:** Kuo-Chuan Hung, Hsiu-Lan Weng, Yi-Chen Lai, I-Wen Chen

**Affiliations:** 1 Department of Anesthesiology, Chi Mei Medical Center, Tainan, Taiwan; 2 School of Medicine, College of Medicine, National Sun Yat-sen University, Kaohsiung, Taiwan; 3 Department of Anesthesiology, E-Da Hospital, I-Shou University, Kaohsiung, Taiwan; 4 Department of Anesthesiology, Chi Mei Medical Center, Liouying, Tainan, Taiwan

**Keywords:** dementia, hearing loss, propensity score matching, sensory impairment, vision impairment, vitamin D deficiency

## Abstract

**Background:**

Sensory impairment is a strong modifiable risk factor for dementia; however, whether vitamin D deficiency (VDD) was associated with increased risk in this high-risk group remains unclear. Individuals with vision or hearing impairment are prone to VDD due to reduced sunlight exposure, making this question clinically relevant.

**Methods:**

From the TriNetX Global Collaborative Network, this retrospective cohort study identified adults aged ≥50 years with documented vision and/or hearing impairment and serum 25-hydroxyvitamin D [25(OH)D] measurement. Patients were classified into VDD group (<20 ng/mL) and control group (≥30 ng/mL). The index date was defined as the date of the first 25(OH)D measurement that met the cohort-specific threshold and satisfied all eligibility criteria. The primary outcome was incident dementia over a 10-year follow-up. Secondary outcomes included dementia subtypes (i.e., vascular dementia and Alzheimer’s disease), cognitive impairment, osteoporotic fracture (positive control), healthcare visits (detection bias assessment), and recurrent VDD.

**Results:**

After propensity score matching, 158,382 patients were included in each cohort. Compared to the control group, the VDD group was associated with a significantly higher risk of incident dementia [hazard ratio (HR), 1.55; *p* < 0.001], vascular dementia (HR, 1.70; *p* < 0.001), Alzheimer’s disease (HR, 1.48; *p* < 0.001), cognitive impairment (HR, 1.40; *p* < 0.001), and subsequent VDD (HR, 4.73; *p* < 0.001). The risk of osteoporotic fracture was significantly associated with VDD (HR, 1.34; *p* < 0.001), whereas healthcare visits were slightly lower in the VDD group (HR, 0.91; *p* < 0.001), arguing against detection bias. Vitamin D insufficiency was associated with a significant but attenuated association with dementia (HR, 1.39; *p* < 0.001).

**Conclusion:**

In this cohort study, VDD was associated with an increased risk of incident dementia in adults with vision and/or hearing impairment, with exploratory findings supportive of a graded pattern. Although residual confounding cannot be excluded, these findings raise the possibility that low vitamin D status may be a potentially modifiable contributor to dementia risk in sensory-impaired populations.

## 1 Introduction

Dementia represents one of the most pressing global health challenges, with over 55 million people currently affected, and projections exceeding 150 million by 2050 (1). In the absence of curative therapies, identifying modifiable risk factors has become a central strategy for dementia prevention (2, 3). Among potentially modifiable risk factors for dementia, sensory impairment has emerged as one of the most significant (4–10). Mechanistically, sensory deprivation may accelerate cognitive decline through reduced environmental stimulation, increased cognitive load, social isolation, and diminished physical activity (11–14). The 2020 Lancet Commission identified hearing loss as the single largest modifiable risk factor for dementia (3), and subsequent evidence has demonstrated that dual sensory impairment is associated with a 160% increased risk of all-cause dementia and up to 267% for Alzheimer’s disease (4).

Experimental and clinical evidence suggests that vitamin D may influence neuroprotection through amyloid-beta clearance, neurotrophin regulation, and anti-inflammatory pathways (15–18). Vitamin D deficiency (VDD) has also been implicated in dementia risk, with meta-analyses reporting a 25–54% increased risk of all-cause dementia (19–21). Notably, adults with sensory impairment may be especially vulnerable to VDD because reduced mobility and less outdoor activity can limit sunlight exposure (22, 23). These observations suggest that VDD may represent a potentially modifiable contributor to dementia risk in adults with sensory impairment. However, this question has not been specifically examined. Because sensory impairment itself is strongly associated with dementia, the incremental association of VDD may be difficult to detect in this high-risk population. Clarifying this issue is clinically relevant, as it may inform whether vitamin D screening and correction should be considered in sensory-impaired adults.

To address this gap, we conducted a large-scale propensity score-matched cohort study of adults aged ≥50 years with documented vision and/or hearing impairment and evaluated whether VDD was associated with an increased risk of incident dementia over 10 years of follow-up.

## 2 Methods

### 2.1 Data source

This retrospective cohort study was conducted using the TriNetX Global Collaborative Network, which includes structured data on demographics, diagnoses (ICD-10-CM codes), procedures, medications, and laboratory measurements. Data are aggregated and anonymized at the source institutional level, and no direct patient identifiers are accessible to investigators. This database has been widely used and validated in numerous cohort studies (24–26). The study protocol was approved by the Institutional Review Board of Chi Mei Medical Center. All procedures were in accordance with the principles of the Declaration of Helsinki.

### 2.2 Study population and inclusion criteria

Adults aged ≥50 years were considered eligible if they had at least one documented serum 25-hydroxyvitamin D [25(OH)D] measurement and a prior diagnosis of vision and/or hearing impairment. Vision impairment was defined using ICD-10-CM codes for blindness and low vision (H54), cataract (H25–H26), macular degeneration (H35.3), and glaucoma (H40–H42). Hearing impairment was defined as conductive or sensorineural hearing loss (H90) or other/unspecified hearing loss (H91). Sensory impairment must have been documented before the index date. Because TriNetX does not consistently provide standardized severity measures, impairment severity could not be reliably ascertained. The sensory-impaired cohort included patients with vision impairment, hearing impairment, or both; dual sensory impairment was not analyzed separately. Cataract was included as part of the broader vision impairment definition and was not interpreted as a direct measure of functional visual severity (27). This broad definition was selected to capture clinically recognized sensory disorders in older adults that are consistently identifiable in large-scale EHR data and have been linked in prior literature to cognitive decline or dementia risk (4, 5, 27–29).

Patients were assigned according to the first recorded 25(OH)D measurement that met the cohort-specific threshold, rather than the very first available 25(OH)D result. Patients entered the VDD cohort at the first 25(OH)D measurement <20 ng/mL and the control cohort at the first measurement ≥30 ng/mL; this date served as the index date. We used the first qualifying 25(OH)D measurement to define baseline vitamin D status because this approach is practical, clinically intuitive, and suitable for large real-world EHR data. Requiring repeated or averaged measurements would have substantially reduced the study population and may have preferentially selected patients undergoing more frequent laboratory testing. To reduce exposure misclassification, patients were excluded if they had a discordant vitamin D value within the 24 months prior to the index measurement (i.e., a value ≥30 ng/mL in the VDD cohort or <20 ng/mL in the control cohort).

### 2.3 Exclusion criteria

Patients with a prior diagnosis of dementia or related neurodegenerative cognitive disorders before the index date were excluded. These exclusions included vascular dementia (ICD-10-CM: F01), dementia in other diseases classified elsewhere (F02), unspecified dementia (F03), Alzheimer’s disease (G30), and other degenerative diseases of the nervous system (G31–G32), including mild cognitive impairment where applicable (e.g., G31.84). Additional exclusion criteria were advanced chronic kidney disease (stages 4–5; N18.4–N18.6), dialysis dependence (Z99.2), and major psychotic or bipolar disorders (F20–F31). Patients with stroke or intracranial injury (I61, I63, S06) within one year before the index date were also excluded, as these acute neurological events are independently associated with vascular dementia and cognitive decline, and their inclusion could confound the association between VDD and incident dementia.

In addition, eligible patients were required to have at least one healthcare encounter between 1 and 5 years after the index date to ensure an observable follow-up period within the EHR system. Because dementia outcomes are ascertained through subsequent clinical documentation, inclusion of patients with no post-index contact could lead to incomplete outcome capture and potential detection bias. A 1-to-5-year window was selected rather than a longer interval to reduce survivorship bias while confirming that patients remained within the healthcare system during the early post-landmark period. Patients who died within 1 year of the index date were excluded using a landmark design, and incident dementia outcomes were assessed beginning 365 days after the index date.

### 2.4 Propensity score matching

Propensity score matching (PSM) was performed using 1:1 greedy nearest-neighbor matching without replacement with a caliper width of 0.1 standard deviations of the logit of the propensity score, according to the default TriNetX implementation. Propensity scores were estimated from baseline variables assessed within 5 years before the index date, including age, sex, race, body mass index, smoking status, comorbidities, dementia-related conditions, baseline vitamin D supplementation, and laboratory covariates. The laboratory covariates included estimated glomerular filtration rate, hemoglobin, albumin, hemoglobin A1c, and C-reactive protein. These biomarkers were selected to reflect renal function, hematologic status, nutritional status, glycemic control, and systemic inflammation, which may be associated with both vitamin D status and dementia risk. Missing laboratory data were not imputed. Covariate balance was assessed using standardized mean differences (SMDs), with values <0.1 considered indicative of adequate balance. Common support was assessed by visual inspection of propensity score density plots before and after matching, which demonstrated substantial overlap between the matched cohorts.

### 2.5 Outcome measures

The primary outcome was incident dementia, comprising vascular dementia (F01), dementia in other diseases (F02), unspecified dementia (F03), and Alzheimer’s disease (G30). Dementia outcomes were identified using prespecified ICD-10-CM diagnosis codes in TriNetX. Incident dementia was defined as the first qualifying diagnosis recorded after the landmark period. Repeated encounters, specialist confirmation, and anti-dementia medication use were not required. Because this study relied on routinely collected EHR data from a federated network, direct chart validation was not feasible.

Secondary outcomes included dementia subtypes (vascular dementia and Alzheimer’s disease), cognitive impairment, healthcare visits, osteoporotic fractures, and recurrent VDD [25(OH)D <20 ng/mL]. Cognitive impairment was evaluated as a distinct secondary endpoint using a separate diagnostic coding framework (G31.84) and was analyzed independently from the primary dementia outcomes. Osteoporotic fractures served as a positive control outcome, given their established association with VDD, while healthcare visits were compared between groups to assess potential detection bias. Recurrent VDD was evaluated to determine whether a single baseline measurement reflected chronic vitamin D status. Outcome ascertainment commenced 365 days after the index date, with follow-up extending up to 10 years. Patients were censored at dementia diagnosis, death, last recorded encounter, or the end of follow-up, whichever occurred first.

### 2.6 Sensitivity, subgroup, and exploratory analysis across vitamin D categories

Three sensitivity analyses were performed: Model I restricted to patients with baseline vision impairment and Model II restricted to those with baseline hearing impairment, and Model III removed the requirement for at least one healthcare encounter between 1 and 5 years after the index date. Model III was designed to address the concern that conditioning cohort eligibility on post-index healthcare engagement may introduce selection or survivorship bias by preferentially retaining patients with more sustained system contact. Pre-specified subgroup analyses examined potential effect modification by age (50–70 vs. >70 years), sex, hypertension, diabetes mellitus, obesity, sleep disorders, and mood disorders. To explore whether the association varied across clinically relevant categories of vitamin D status, an additional propensity score-matched analysis compared patients with vitamin D insufficiency [25(OH)D 20–30 ng/mL] against those with sufficient levels (≥30 ng/mL). By assessing whether the association was attenuated in the insufficiency group relative to the primary deficiency cohort (<20 ng/mL), this analysis provided supportive evidence of a graded association across vitamin D categories. This categorical comparison was exploratory and was not intended to represent a formal continuous dose–response analysis.

### 2.7 Statistical analysis

Cox proportional hazards models were used to estimate hazard ratios (HRs) with 95% confidence intervals (CIs) for all time-to-event outcomes. Outcome-free probability was illustrated using Kaplan–Meier curves. The proportional hazards assumption was evaluated using Schoenfeld residual tests. A multivariate Cox regression model was constructed to incorporate baseline covariates and assess the independent association between VDD and dementia. No adjustment for multiple comparisons was applied, as outcomes were hierarchically structured with a single pre-specified primary endpoint, while secondary and control outcomes served confirmatory or validation roles. All tests were two-sided, with a significance level set at *p* < 0.05.

## 3 Results

### 3.1 Patient selection and baseline characteristics

**Figure 1:**
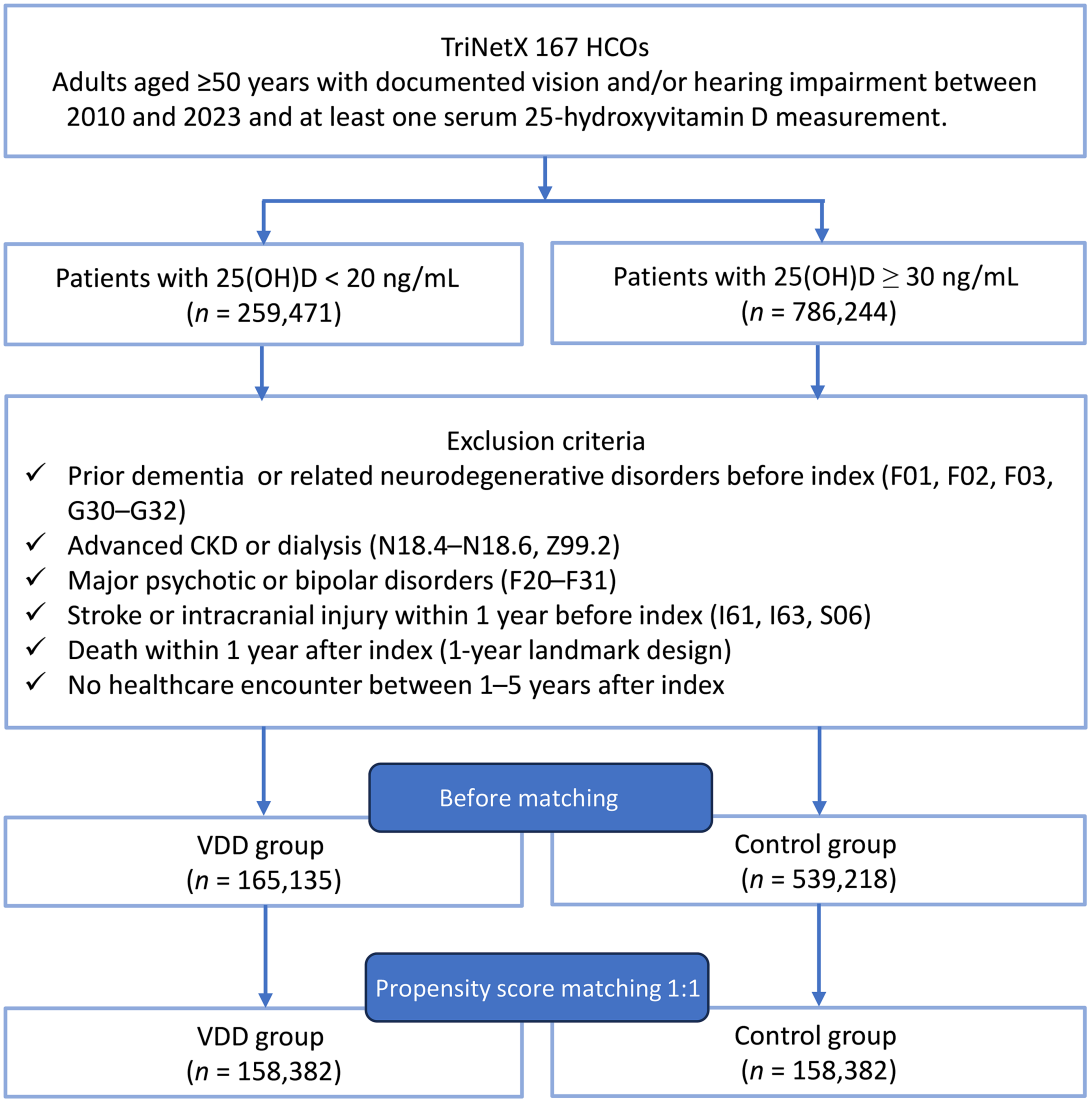
Patient selection flowchart. Adults aged ≥50 years with at least one serum 25-hydroxyvitamin D [25(OH)D] measurement and a documented diagnosis of vision and/or hearing impairment were identified using the TriNetX Global Collaborative Network (2010–2023). Patients were assigned to either the vitamin D deficiency (VDD) cohort [25(OH)D <20 ng/mL] or the control cohort [25(OH)D ≥30 ng/mL]. Sequential exclusion criteria were applied, followed by 1:1 propensity score matching. VDD, vitamin D deficiency; 25(OH)D, 25-hydroxyvitamin D; HCO, healthcare organization; CKD, chronic kidney disease; PSM, propensity score matching.

**Figure 2:**
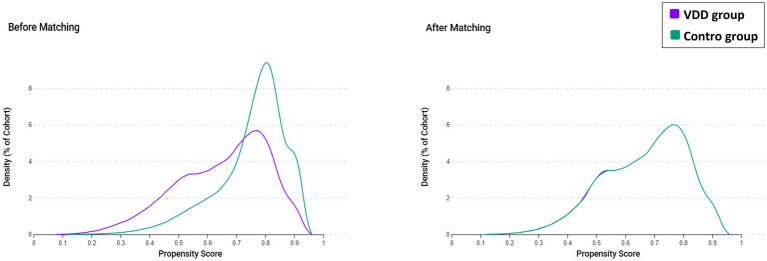
Propensity score density plots before and after matching. The left panel displays the distribution of propensity scores in the vitamin D deficiency and control cohorts before matching, demonstrating substantial overlap with some imbalance. The right panel displays the propensity score distributions after 1:1 matching, showing near-complete overlap between cohorts, indicating adequate covariate balance. VDD, vitamin D deficiency.

Before matching, 165,135 patients were identified in the VDD cohort and 539,218 in the control cohort, respectively (Figure 1). Substantial baseline imbalances were observed, with the VDD group being younger (63.8 vs. 66.9 years), less frequently White (54.3% vs. 74.6%), and having a higher prevalence of diabetes mellitus (34.0% vs. 24.3%), obesity (29.9% vs. 22.1%), and nicotine dependence (14.6% vs. 8.1%) (Table 1). Before matching, laboratory data availability in the VDD cohort was approximately 85.5% for estimated glomerular filtration rate, 82.5% for hemoglobin, 76.8% for albumin, 54.8% for hemoglobin A1c, and 20.6% for C-reactive protein, with similar proportions in the comparator cohort. After matching, 158,382 patients remained in each cohort, with all standardized mean differences below 0.1, indicating adequate covariate balance. Propensity score density plots confirmed a near-complete distributional overlap between cohorts after matching (Figure 2).

**Table 1:** Baseline characteristics of patients with vitamin D deficiency and vitamin D sufficiency.

Variables	Before matching			After matching		
	VDD group (*n* = 165,135)	Control group (*n* = 539,218)	SMD	VDD group (*n* = 158,382)	Control group (*n* = 158,382)	SMD
Patient characteristics						
Age at index (years)	63.8 ± 11.8	66.9 ± 10.7	0.272	64.2 ± 11.8	64.3 ± 11.0	0.013
Female	110,039 (66.6)	384,834 (71.4)	0.102	106,502 (67.2)	106,479 (67.2)	0.000
BMI ≥30 kg/m^2^	69,192 (41.9)	175,785 (32.6)	0.193	64,620 (40.8)	66,204 (41.8)	0.022
White	89,636 (54.3)	402,307 (74.6)	0.435	89,119 (56.3)	86,408 (54.6)	0.034
Black or African American	42,465 (25.7)	65,963 (12.2)	0.349	37,927 (23.9)	38,715 (24.4)	0.012
Asian	6,381 (3.9)	25,606 (4.7)	0.044	6,369 (4.0)	6,490 (4.1)	0.004
Comorbidities						
Factors influencing health status and contact with health services	123,952 (75.1)	411,697 (76.4)	0.030	118,752 (75.0)	119,117 (75.2)	0.005
Essential (primary) hypertension	102,888 (62.3)	309,059 (57.3)	0.102	97,369 (61.5)	99,137 (62.6)	0.023
Diabetes mellitus	56,160 (34.0)	131,096 (24.3)	0.215	51,641 (32.6)	52,593 (33.2)	0.013
Overweight and obesity	49,306 (29.9)	119,161 (22.1)	0.178	45,579 (28.8)	46,483 (29.3)	0.013
Sleep disorders	41,448 (25.1)	126,671 (23.5)	0.037	39,164 (24.7)	39,831 (25.1)	0.010
Anxiety disorders	40,552 (24.6)	127,078 (23.6)	0.023	38,560 (24.3)	39,080 (24.7)	0.008
Mood disorders	39,671 (24.0)	108,349 (20.1)	0.095	37,096 (23.4)	37,339 (23.6)	0.004
Ischemic heart diseases	28,513 (17.3)	81,207 (15.1)	0.060	26,685 (16.8)	27,032 (17.1)	0.006
Nicotine dependence	24,101 (14.6)	43,721 (8.1)	0.206	21,054 (13.3)	21,256 (13.4)	0.004
Chronic kidney disease (CKD)	19,831 (12.0)	55,413 (10.3)	0.055	18,447 (11.6)	18,699 (11.8)	0.005
Diseases of liver	17,342 (10.5)	44,857 (8.3)	0.075	16,142 (10.2)	16,384 (10.3)	0.005
COPD	16,674 (10.1)	39,391 (7.3)	0.099	15,160 (9.6)	15,501 (9.8)	0.007
Heart failure	14,337 (8.7)	31,854 (5.9)	0.107	12,848 (8.1)	12,946 (8.2)	0.002
Cerebrovascular diseases	12,335 (7.5)	36,661 (6.8)	0.026	11,600 (7.3)	11,800 (7.5)	0.005
Atrial fibrillation and flutter	11,886 (7.2)	38,912 (7.2)	0.001	11,288 (7.1)	11,338 (7.2)	0.001
Major depressive disorder	9,499 (5.8)	25,010 (4.6)	0.050	8,801 (5.6)	8,789 (5.5)	0.000
Systemic connective tissue disorders	6,371 (3.9)	22,744 (4.2)	0.018	6,140 (3.9)	6,235 (3.9)	0.003
Alcohol related disorders	6,940 (4.2)	12,653 (2.3)	0.104	5,950 (3.8)	5,896 (3.7)	0.002
COVID-19	5,036 (3.1)	17,396 (3.2)	0.010	4,839 (3.1)	4,843 (3.1)	0.000
Malnutrition	3,507 (2.1)	7,447 (1.4)	0.057	3,128 (2.0)	3,251 (2.1)	0.006
Laboratory data						
Hemoglobin ≥12 g/dL	126,082 (76.4)	389,291 (72.2)	0.095	120,183 (75.9)	121,393 (76.6)	0.018
Albumin ≥3.5 g/dL	36,808 (22.3)	80,211 (14.9)	0.191	33,169 (20.9)	33,578 (21.2)	0.006
eGFR ≥60 mL/min/1.73m^2^	44,006 (26.6)	121,241 (22.5)	0.097	41,097 (25.9)	42,007 (26.5)	0.013
CRP ≥10 mg/L	16,722 (10.1)	34,321 (6.4)	0.137	15,017 (9.5)	15,132 (9.6)	0.002
Hemoglobin A1c ≥9%	18,015 (10.9)	27,226 (5.0)	0.218	15,289 (9.7)	15,395 (9.7)	0.002
Medication						
Central nervous system medications	127,962 (77.5)	392,261 (72.7)	0.110	121,573 (76.8)	122,535 (77.4)	0.014
Cardiovascular medications	123,115 (74.6)	385,604 (71.5)	0.069	117,028 (73.9)	118,266 (74.7)	0.018
Benzodiazepine derivative sedatives/hypnotics	65,180 (39.5)	203,834 (37.8)	0.034	61,782 (39.0)	62,643 (39.6)	0.011
Blood glucose-lowering drugs	38,551 (23.3)	89,944 (16.7)	0.167	35,337 (22.3)	35,856 (22.6)	0.008
Vitamin D supplementation	32,099 (19.4)	158,962 (29.5)	0.235	31,884 (20.1)	35,441 (22.4)	0.055
Insulins and analogues	29,343 (17.8)	57,523 (10.7)	0.204	25,921 (16.4)	26,436 (16.7)	0.009
Iron supplementation	14,046 (8.5)	34,150 (6.3)	0.083	12,820 (8.1)	13,075 (8.3)	0.006

Data are presented as mean ± standard deviation for continuous variables and *n* (%) for categorical variables. VDD, vitamin D deficiency; 25(OH)D, 25-hydroxyvitamin D; BMI, body mass index; CKD, chronic kidney disease; COPD, chronic obstructive pulmonary disease; eGFR, estimated glomerular filtration rate; CRP, C-reactive protein; HbA1c, hemoglobin A1c; SMD, standardized mean difference.

### 3.2 Association between VDD and 10-year outcomes

**Figure 3:**
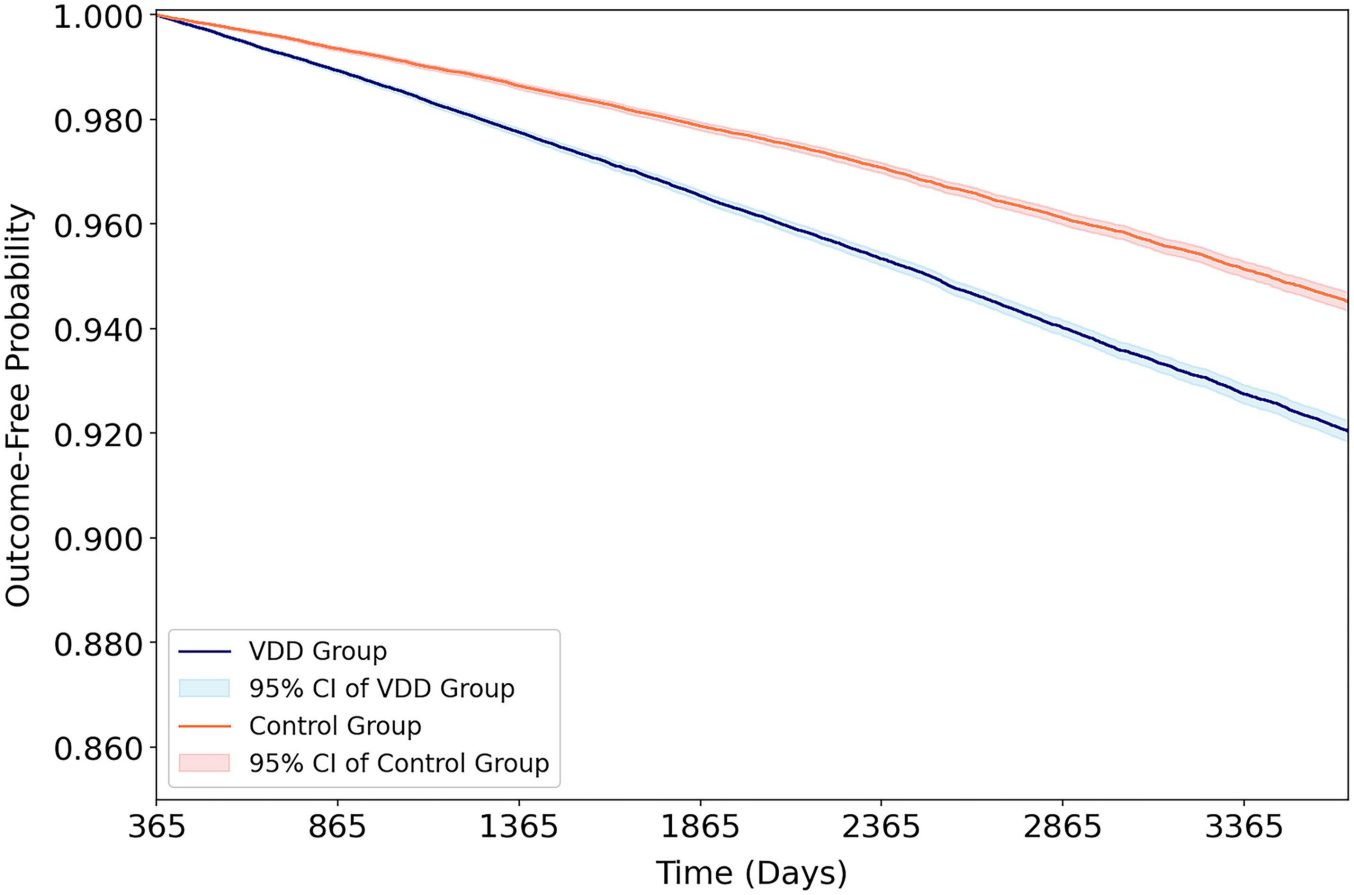
Kaplan–Meier outcome-free probability curves for incident dementia in the vitamin D deficiency and control cohorts over the 10-year follow-up period. Outcome assessment commenced 365 days after the index date using a landmark approach. The vitamin D deficiency cohort demonstrated a progressively lower probability of remaining dementia-free compared with the control cohort throughout the follow-up. Shaded areas represent 95% confidence intervals. VDD, vitamin D deficiency; HR, hazard ratio; CI, confidence interval.

Over a maximum follow-up of 10 years after the 1-year landmark period, VDD was associated with a significantly higher risk of dementia than vitamin D sufficiency (HR 1.55, *p* < 0.001) (Table 2 and Figure 3). Among dementia subtypes, VDD was associated with both vascular dementia (HR 1.70, *p* < 0.001) and Alzheimer’s disease (HR 1.48, *p* < 0.001). Cognitive impairment was also more frequent in the VDD cohort (HR 1.40, *p* < 0.001). The positive control outcome, osteoporotic fracture, demonstrated a significant association with VDD (HR 1.34, *p* < 0.001), supporting the biological plausibility of the exposure classification. Healthcare visits were slightly lower in the VDD group (HR 0.91, *p* < 0.001), and patients with baseline VDD had a markedly higher risk of subsequent VDD during follow-up (HR 4.73, *p* < 0.001).

**Table 2:** Association between vitamin D deficiency and dementia risk during the 10-year follow-up (*n* = 158,382 per group).

Outcomes	VDD group events (%)	Control group events (%)	HR (95% CI)	*p*-value	ARD
Dementia	7,152 (4.52%)	4,544 (2.87%)	1.55 (1.49–1.61)	<0.001	1.65%
Vascular dementia	1,433 (0.91%)	821 (0.52%)	1.70 (1.56–1.85)	<0.001	0.39%
Alzheimer’s disease	1,998 (1.26%)	1,317 (0.83%)	1.48 (1.38–1.59)	<0.001	0.43%
Cognitive impairment	3,551 (2.24%)	2,479 (1.57%)	1.40 (1.33–1.48)	<0.001	0.68%
Healthcare visits	158,264 (99.93%)	158,265 (99.93%)	0.91 (0.90–0.92)	<0.001	0.00%
Osteoporotic fracture	2,842 (1.79%)	2,083 (1.32%)	1.34 (1.26–1.42)	<0.001	0.48%
VDD risk	54,318 (34.30%)	13,915 (8.79%)	4.73 (4.65–4.82)	<0.001	25.51%

Values are presented as the number of events (percentage). VDD, vitamin D deficiency; ARD, absolute risk difference; HR, hazard ratio; CI, confidence interval.

### 3.3 Sensitivity and subgroup analyses

**Figure 4:**
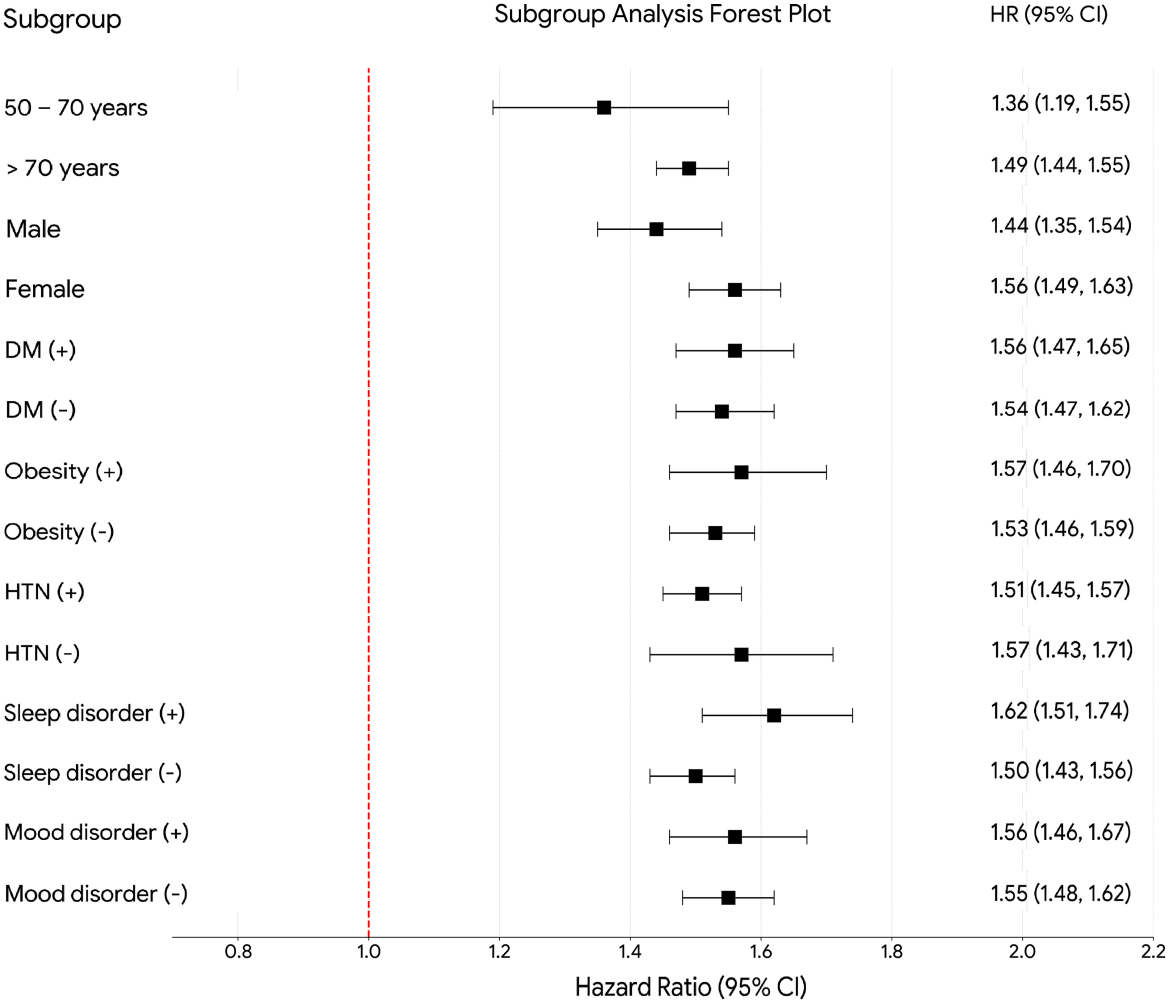
Forest plot of subgroup analyses for the association between vitamin D deficiency and incident dementia. Hazard ratios with 95% confidence intervals are presented for the prespecified subgroups defined by age (50–70 vs. >70 years), sex (male vs. female), hypertension (HTN; yes vs. no), diabetes mellitus (DM; yes vs. no), obesity (yes vs. no), sleep disorders (yes vs. no), and mood disorders (yes vs. no). All subgroup analyses were conducted in a propensity score-matched cohort. VDD, vitamin D deficiency; HR, hazard ratio; CI, confidence interval.

All three sensitivity analyses yielded results consistent with the primary analysis (Table 3). The association between VDD and dementia persisted when restricting to patients with baseline vision impairment (Model I: HR 1.51, *p* < 0.001), those with baseline hearing impairment (Model II: HR 1.67, *p* < 0.001), and when removing the post-index healthcare encounter requirement (Model III: HR 1.53, *p* < 0.001). In Model III, the associations with vascular dementia (HR 1.62, *p* < 0.001), Alzheimer’s disease (HR 1.45, *p* < 0.001), and cognitive impairment (HR 1.41, *p* < 0.001) remained directionally consistent with the primary findings. Subgroup analyses demonstrated a consistent association between VDD and dementia across all prespecified strata (Figure 4). The association was significant regardless of age group (50–70 vs. >70 years), sex, hypertension, diabetes mellitus, obesity, sleep disorders, and mood disorders.

**Table 3:** Sensitivity analyses for the association between vitamin D deficiency and dementia risk during the 10-year follow-up.

Outcomes	Model I			Model II			Model III		
	HR (95% CI)	*p*-value	ARD	HR (95% CI)	*p*-value	ARD	HR (95% CI)	*p*-value	ARD
Dementia	1.51 (1.45–1.57)	<0.001	1.61%	1.67 (1.57–1.77)	<0.001	1.91%	1.53 (1.47–1.59)	<0.001	1.51%
Vascular dementia	1.67 (1.52–1.84)	<0.001	0.40%	1.89 (1.64–2.19)	<0.001	0.44%	1.62 (1.49–1.76)	<0.001	0.34%
Alzheimer’s disease	1.38 (1.28–1.49)	<0.001	0.37%	1.63 (1.45–1.82)	<0.001	0.53%	1.45 (1.35–1.55)	<0.001	0.39%
Cognitive impairment	1.37 (1.29–1.45)	<0.001	0.65%	1.38 (1.27–1.49)	<0.001	0.67%	1.41 (1.34–1.48)	<0.001	0.64%
Healthcare visits	0.91 (0.90–0.92)	<0.001	0.00%	0.92 (0.91–0.93)	<0.001	−0.03%	0.90 (0.90–0.91)	<0.001	−1.10%
Osteoporotic fracture	1.35 (1.26–1.44)	<0.001	0.51%	1.36 (1.24–1.49)	<0.001	0.49%	1.41 (1.33–1.49)	<0.001	0.51%
VDD risk	4.58 (4.48–4.67)	<0.001	26.04%	5.63 (5.44–5.82)	<0.001	25.94%	4.76 (4.67–4.85)	<0.001	24.33%

Model I included patients with baseline vision impairment (*n* = 116,749 per group). Model II included patients with baseline hearing impairment (*n* = 59,536 per group). Model III removed the requirement for at least one healthcare encounter between 1 and 5 years after the index date (*n* = 165,830). HR, hazard ratio; CI, confidence interval; VDD, vitamin D deficiency; ARD, absolute risk difference.

### 3.4 Multivariate Cox regression analysis for predictors of dementia

In the multivariate Cox regression model (Table 4), VDD remained independently associated with dementia after adjusting for baseline covariates (HR 1.52, *p* < 0.001). Other significant predictors of dementia were older age (HR 1.13 per year, *p* < 0.001), mood disorders (HR 1.51, *p* < 0.001), diabetes mellitus (HR 1.35, *p* < 0.001), malnutrition (HR 1.42, *p* < 0.001), cerebrovascular diseases (HR 1.37, *p* < 0.001), nicotine dependence (HR 1.32, *p* < 0.001), and alcohol-related disorders (HR 1.30, *p* < 0.001).

**Table 4:** Multivariate Cox regression analysis for predictors of incident dementia in the propensity score-matched cohort.

Variable	Hazard ratio (95% CI)	*p*-value
VDD vs. control group	1.52 (1.48–1.56)	<0.001
Male	1.10 (1.07–1.14)	<0.001
Age at index	1.13 (1.13–1.13)	<0.001
Essential (primary) hypertension	1.18 (1.14–1.21)	<0.001
Diabetes mellitus	1.35 (1.32–1.39)	<0.001
Overweight and obesity	0.94 (0.91–0.98)	0.001
Sleep disorders	1.03 (1.00–1.07)	0.043
Mood (affective) disorders	1.51 (1.46–1.56)	<0.001
Anxiety and related disorders	1.19 (1.15–1.23)	<0.001
Ischemic heart diseases	1.14 (1.11–1.18)	<0.001
Nicotine dependence	1.32 (1.26–1.38)	<0.001
Chronic kidney disease (CKD)	1.12 (1.08–1.16)	<0.001
Diseases of liver	0.99 (0.94–1.04)	0.626
Cerebrovascular diseases	1.37 (1.32–1.42)	<0.001
Major depressive disorder	1.22 (1.15–1.30)	<0.001
Alcohol related disorders	1.30 (1.20–1.40)	<0.001
Malnutrition	1.42 (1.31–1.54)	<0.001

VDD, vitamin D deficiency; CKD, chronic kidney disease; HR, hazard ratio; CI, confidence interval.

### 3.5 Exploratory analysis across vitamin D categories

The exploratory analysis comparing vitamin D insufficiency [25(OH)D 20–30 ng/mL] with sufficiency (≥30 ng/mL) revealed a significant but attenuated association with dementia (HR 1.39, *p* < 0.001) (Table 5), compared with the primary analysis of VDD (<20 ng/mL; HR 1.55). This graded pattern, in which the magnitude of association was smaller in the insufficiency group than in the deficiency group, is supportive of a graded association across vitamin D categories.

**Table 5:** Association between vitamin D insufficiency and dementia risk during the 10-year follow-up (*n* = 278,251 per group).

Outcomes	VDI group events (%)	Control group events (%)	HR (95% CI)	*p*-value	ARD
Dementia	11,614 (4.17%)	8,263 (2.97%)	1.39 (1.35–1.43)	<0.001	1.20%
Vascular dementia	2,272 (0.82%)	1,445 (0.52%)	1.54 (1.44–1.64)	<0.001	0.30%
Alzheimer’s disease	3,544 (1.27%)	2,437 (0.88%)	1.42 (1.35–1.50)	<0.001	0.40%
Cognitive impairment	6,391 (2.30%)	4,736 (1.70%)	1.33 (1.28–1.38)	<0.001	0.60%
Healthcare visits	278,079 (99.94%)	278,066 (99.93%)	0.96 (0.96–0.97)	0.009	0.01%
Osteoporotic fracture	5,417 (1.95%)	3,907 (1.40%)	1.36 (1.31–1.42)	<0.001	0.54%
VDD risk	45,512 (16.36%)	20,640 (7.42%)	2.33 (2.29–2.36)	<0.001	8.94%

Vitamin D insufficiency (VDI) was defined as 25-hydroxyvitamin D [25(OH)D] 20–30 ng/mL, and control group as 25(OH)D ≥30 ng/mL. VDI, vitamin D insufficiency; 25(OH)D, 25-hydroxyvitamin D; ARD, absolute risk difference; HR, hazard ratio; CI, confidence interval; VDD, vitamin D deficiency.

## 4 Discussion

In this large-scale propensity score-matched cohort study of adults with documented vision and/or hearing impairment, VDD was associated with a significantly elevated risk of incident dementia over a 10-year follow-up period compared to vitamin D sufficiency. This association extended to dementia subtypes, including vascular dementia and Alzheimer’s disease, as well as cognitive impairment. The findings remained consistent across sensitivity analyses restricted to vision or hearing impairment alone, when the post-index healthcare encounter requirement was removed, across all prespecified subgroups, and after multivariate adjustment. An exploratory graded pattern was observed, with vitamin D insufficiency conferring a lesser but still significant risk elevation compared with frank deficiency. Notably, the positive control outcome supported the biological plausibility of the exposure–outcome relationship, whereas analyses of healthcare utilization mitigated concerns regarding detection bias.

Although accumulating evidence has linked VDD to dementia risk in the general population (19–21), the clinical relevance of this association in individuals with sensory impairment remains unexplored. Vision and hearing impairments are recognized as the most significant modifiable risk factors for dementia (4–10), and the risk they confer may substantially exceed that attributed to VDD alone. Given this high baseline risk, any incremental effect of VDD may be minimal or obscured by the dominant impact of sensory deprivation on cognitive decline. Our findings suggest that VDD was associated with a statistically significant and directionally consistent increase in dementia risk even within this high-risk population, although residual confounding by unmeasured factors such as physical activity, diet, and sunlight exposure cannot be excluded. Moreover, the association extended to both vascular dementia and Alzheimer’s disease subtypes, as well as cognitive impairment, suggesting that the relationship is not confined to a single pathological pathway. These results may be clinically relevant because individuals with vision or hearing impairments are disproportionately susceptible to VDD owing to reduced outdoor mobility and limited sunlight exposure (22, 23); however, they are rarely targeted for routine vitamin D screening. Whether this observed association reflects a causal relationship that could be modified through screening or supplementation remains to be established. To our knowledge, limited data exist on whether VDD retains prognostic significance specifically in adults with sensory impairment, and this study contributes to addressing this gap.

A notable finding of the present study was the significant association between VDD and vascular dementia. This association remained directionally consistent across sensitivity analyses, supporting the robustness of the observed subtype-specific signal. From a mechanistic perspective, vitamin D deficiency has been linked to endothelial dysfunction, vascular inflammation, oxidative stress, and impaired regulation of vascular tone, all of which may contribute to cerebrovascular injury and the development of vascular cognitive impairment (30–32). Accordingly, the observed association with vascular dementia may be biologically plausible. Nevertheless, this finding should be interpreted with caution, because the observational nature of the study precludes causal inference and residual confounding cannot be entirely ruled out.

Several design features were intended to improve exposure specificity and reduce misclassification. First, we incorporated osteoporotic fractures as a positive control outcome to verify that the vitamin D exposure classification captured a biologically meaningful difference between groups. The consistent association between VDD and osteoporotic fractures across the primary and sensitivity analyses supports the validity of our exposure ascertainment strategy. Second, we evaluated healthcare visits as a proxy for detection bias. The slightly lower visit frequency observed in the VDD cohort indicates that the elevated dementia risk was not attributable to increased surveillance or more frequent clinical encounters among vitamin D-deficient patients. Third, we assessed the subsequent risk of recurrent VDD during follow-up and found that patients with baseline VDD had a markedly higher probability of experiencing later deficiency. This finding was directionally consistent with relative stability of vitamin D status over time. However, it should be interpreted as supportive evidence of exposure stability rather than as independent validation that a single qualifying 25(OH)D measurement definitively reflects chronic exposure.

Multivariate Cox regression analysis revealed that VDD remained independently associated with dementia after adjusting for a comprehensive set of baseline covariates. Notably, the risk factors identified as significant predictors of dementia in this sensory-impaired cohort—including advancing age, diabetes mellitus, cerebrovascular disease, mood disorders, malnutrition, nicotine dependence, and alcohol-related disorders—are well-established determinants of dementia in the general population (33). The preservation of these conventional risk factor associations within a high-risk sensory-impaired population suggests that known dementia risk factors retain their prognostic significance even in the presence of dominant sensory-related contributors. This observation supports the concept that dementia risk in sensory-impaired individuals is shaped by the same multifactorial framework applicable to broader populations, with VDD representing an additional, potentially modifiable component.

Although VDD was associated with a statistically significant increase in the hazard of incident dementia, the absolute risk difference was modest. Accordingly, the clinical significance of this finding should be interpreted with caution, particularly given the observational design and the potential for residual confounding by factors closely related to vitamin D status, including physical activity, sunlight exposure, dietary patterns, and frailty. Moreover, given that excessive vitamin D supplementation may pose adverse health risks, these findings should not be interpreted as evidence supporting routine high-dose supplementation without appropriate clinical evaluation. In addition, the generalizability of our findings may be limited by the characteristics of the TriNetX network, which reflects patients receiving care within participating healthcare organizations rather than the general population. Therefore, these results may not be directly applicable to populations with different demographic structures, healthcare access, clinical practices, or patterns of vitamin D testing and supplementation.

The exploratory analysis across vitamin D categories provides additional contextual support for the observed association between low vitamin D status and dementia risk. Compared with the primary analysis, in which VDD was associated with a higher risk magnitude, the vitamin D insufficiency group demonstrated a significant but attenuated association with incident dementia. This pattern is supportive of a graded association across clinically defined vitamin D categories. However, because this comparison was based on separate categorical matched analyses rather than continuous exposure modeling, it should be interpreted cautiously and not as definitive evidence of a biological dose–response relationship. Nevertheless, the directional consistency of the findings is compatible with, but does not confirm, the biological plausibility of the observed association. The persistence of significant associations for the positive control outcome and the absence of increased healthcare visits in the insufficiency group further reinforce the robustness of these findings.

Our operational definition of sensory impairment was intentionally broad and included heterogeneous visual and hearing disorders with differing severity, chronicity, reversibility, and biological mechanisms. Although this pragmatic EHR-based approach enabled identification of a clinically relevant at-risk population, it may have reduced phenotypic specificity and limited the interpretability of the pooled sensory-impairment analysis. To address this, we performed sensitivity analyses separately for visual impairment and hearing impairment, which yielded generally consistent results. However, we did not conduct a dedicated analysis of dual sensory impairment because the number of patients meeting this phenotype was limited, and this subgroup analysis was not part of the original analytical plan. Future studies focused specifically on dual sensory impairment are warranted.

Several limitations warrant consideration. First, several important determinants of both vitamin D status and dementia risk—such as physical activity, sunlight exposure, diet, education, socioeconomic status, frailty-related behaviors, and non-prescribed supplementation—are not well captured in TriNetX. Consequently, residual confounding may persist, and the observed associations should be interpreted with caution. Second, Vitamin D status was based on a single qualifying 25(OH)D measurement and may therefore be affected by seasonal variation, supplementation, acute illness, and other time-varying influences. Site-level differences in laboratory calibration across TriNetX organizations also could not be directly assessed. Third, dementia, its subtypes, and cognitive impairment were identified using ICD-10-CM codes within the TriNetX database rather than neuropsychological testing or specialist-confirmed clinical adjudication. In addition, we did not independently validate these diagnoses through chart review, repeated-encounter requirements, or medication confirmation. Accordingly, some degree of outcome misclassification is possible. Fourth, several laboratory variables were incompletely captured in the HER. As a result, residual confounding related to incompletely measured metabolic or inflammatory status cannot be excluded. In addition, post-matching effect estimates were generated using the standard TriNetX analytics pipeline, which does not allow user-level customization of variance estimation. This should be recognized as a methodological limitation of platform-based EHR analyses. Finally, mortality is an important competing event in dementia research. Because our primary objective was etiologic rather than prognostic, we used Cox proportional hazards models to estimate cause-specific hazard associations. However, Kaplan–Meier-based cumulative incidence may overestimate the absolute probability of dementia when competing mortality is substantial, and this limitation should be considered when interpreting our findings.

## 5 Conclusion

In this large propensity score-matched cohort study, VDD was associated with an increased risk of incident dementia in adults with vision and/or hearing impairment, with additional exploratory findings supportive of a graded association across vitamin D categories. These findings raise the possibility that low vitamin D status may be a potentially modifiable contributor to dementia risk in sensory-impaired populations, although residual confounding by unmeasured lifestyle and environmental factors cannot be excluded. These results are hypothesis-generating and should be interpreted within the constraints of the observational study design. Future prospective studies and randomized controlled trials are warranted to determine whether this association is causal and whether targeted vitamin D screening and appropriate supplementation can reduce dementia risk in this population.

## Data Availability

The raw data supporting the conclusions of this article will be made available by the authors, without undue reservation.
